# Colorectal cancer detected by liquid biopsy 2 years prior to clinical diagnosis in the HUNT study

**DOI:** 10.1038/s41416-023-02337-4

**Published:** 2023-07-12

**Authors:** Siv S. Brenne, Poul Henning Madsen, Inge Søkilde Pedersen, Kristian Hveem, Frank Skorpen, Henrik Bygum Krarup, Guro F. Giskeødegård, Eivor A. Laugsand

**Affiliations:** 1 Department of Surgery, Levanger Hospital, Nord-Trøndelag Health Trust, Levanger, Norway; 2 Department of Public Health and Nursing, Norwegian University of Science and Technology, NTNU, N-7489 Trondheim, Norway; 3 Molecular Diagnostics, Aalborg University Hospital, Aalborg, Denmark; 4 Clinical Cancer Research Centre, Aalborg University Hospital, Aalborg, Denmark; 5 Department of Clinical Medicine, Aalborg University, Aalborg, Denmark; 6 Department of Clinical and Molecular Medicine, Norwegian University of Science and Technology, NTNU, N-7489 Trondheim, Norway

**Keywords:** Diagnostic markers, Colorectal cancer, Colorectal cancer

## Abstract

**Background:**

Colorectal cancer (CRC) is often diagnosed in advanced stages. Circulating tumour DNA (ctDNA) has been proposed as an early diagnostic biomarker. However, as a screening tool, ctDNA has mainly been studied in selected populations at the time of clinical diagnosis. The aim of this study was to detect CRC by known ctDNA markers up to 2 years prior to clinical diagnosis.

**Methods:**

In this case–control study, methylated ctDNA markers were detected in plasma samples from 106 healthy controls and 106 individuals diagnosed with CRC within 24 months following participation in The Trøndelag Health Study.

**Results:**

The most specific single markers were *BMP3, FLI1, IKZF1, SFRP1, SFRP2, NPTX2, SLC8A1* and *VIM* (specificity >70%). When combining these into a panel, the CRC sensitivity was 43% (95% CI 42.7–43.4) and the CRC specificity was 86% (95% CI 85.7–86.2). The findings were reproduced in an independent validation set of samples.

**Conclusions:**

Detection of known methylated ctDNA markers of CRC is possible up to 2 years prior to the clinical diagnosis in an unselected population resembling the screening setting. This study supports the hypothesis that some patients could be diagnosed earlier, if ctDNA detection was part of the CRC screening programme.

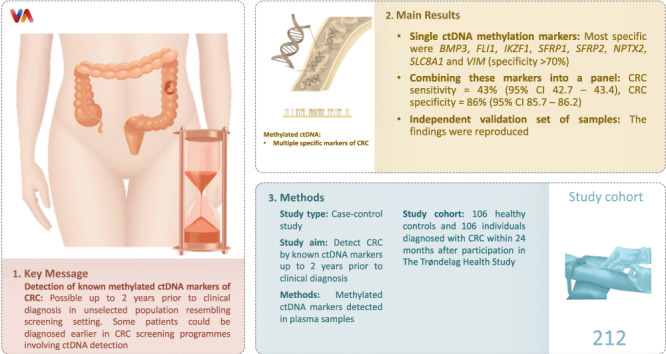

## Background

Colorectal cancer (CRC) is one of the most common malignancies worldwide. The incidence of CRC in Norway is one of the world’s highest with 83.8/100.000 new cases in 2021 and still increasing [1]. CRC is often sporadic and develops over a decade from precursor lesions (the adenoma–carcinoma sequence) [2]. Approximately 15% of patients with CRC are diagnosed with local disease (Stage I), 50% are diagnosed with regionally spread disease (Stage II and III), 25% are diagnosed with distantly spread disease (Stage IV) and for 8% the stage remains unknown [3]. For patients with CRC diagnosed at late stages, treatment options are significantly reduced. The 5-year survival rate ranges from greater than 90% in patients with Stage I disease to slightly above 10% in patients with Stage IV disease [1]. Screening has reduced both incidence and mortality, and more patients have been diagnosed at earlier stages [3]. In Norway, a national screening programme has been decided upon, but is not implemented yet. From a patient’s perspective, earlier detection of CRC, allowing for potentially curable treatment to more patients and less comprehensive treatment regimes, is highly desirable.

Established tools for CRC screening are FIT (Faecal immunochemical test) (sensitivity 74%, specificity 96%), HSgFOBT (High-sensitivity guaiac-based faecal occult blood test) (sensitivity 70%, specificity 93%), colonoscopy (sensitivity 95%, specificity 86%) and CTC (Computed tomographic colonography) (sensitivity 84%, specificity 88%) [4]. Although population-based CRC screening is strongly recommended, participation rates are still far from the desired [5]. Unpleasantness, discomfort and perceived risk with performing the existing screening tests have been identified as screening barriers [5]. Hence, much research in colorectal cancer has focused on earlier diagnosis and screening by less invasive tools, and a series of blood-based biomarkers for CRC has been identified [6].

Circulating tumour DNA (ctDNA) has shown promising results as liquid biopsy for diagnosis, treatment and follow-up of colorectal cancer [6, 7]. ctDNA is the small portion of DNA released into the circulation from tumour cells, among the much larger portion of cell-free DNA (cfDNA) released into the circulation by all other cells [8]. By sensitive methods, it is now possible to detect ctDNA in the plasma and serum of patients with CRC at all stages, as well as in patients with adenomas [6, 7, 9]. ctDNA has been detected in subjects up to 4 years prior to CRC diagnosis, however this study was prospective, had few cases (*n* = 4) and focused on the detection of several cancers simultaneously [10]. Luo et al. detected 19 of 21 CRC cases with a single methylated ctDNA marker in a selected group in another prospective study [11]. The methylation of cytosine to form 5-methylcytosine is a frequent epigenetic modification of the DNA in humans. Increased CpG methylation in promoter regions of genes, especially at CpG-rich sequences termed CpG islands, is associated with transcription repression [12]. Methylation in tumour suppressor genes, genes regulating mitosis and DNA repair is considered an early event in CRC tumourigenesis [12]. ctDNA can be analysed for such aberrant methylation by highly sensitive and specific methods, and analysis of methylated ctDNA is promising as a liquid biopsy biomarker, providing a minimally invasive method for detection, characterisation, prognosis and follow-up of CRC [7].

Approximately 70 methylated promoter regions have been identified as possible blood or stool-based biomarkers for CRC [13]. Some biomarkers are already commercialised as tests for early detection of CRC, Epi proColon 2.0 (sensitivity 66–81% and specificity 96–99%) [14], ColoSure® (sensitivity 38–88%, specificity 82–90%) [12] and Cologuard® (sensitivity 92%, specificity 87%) [14]. Multitarget stool DNA test (mt-sDNA) was implemented in 2018 by the American Cancer Society guidelines, as an alternative for screening for average-risk people [14]. However, in screening subjects, the sensitivity for detecting advanced adenomas was only 9.6% [15]. It now seems that a panel of markers may be necessary to reach the sensitivity and specificity levels required for screening tools. Assays combining methylated ctDNA markers in a panel (such as *APC, MGMT, RASSF2A* and *WIF1*) have reached sensitivities and specificities above 90% [13].

To date, studies investigating ctDNA and its diagnostic ability as a screening tool have mainly been performed in selected populations, including cases at the time of diagnosis (i.e., subjects showing up for screening by colonoscopy/FIT/iFOBT or pre-treatment by surgery/chemotherapy etc.) and matching healthy controls either being colonoscopy negative or self-reported healthy. To our knowledge, no studies have so far systematically searched for methylated ctDNA in blood plasma prior to the clinical diagnosis of CRC in unselected populations, such as in cross-sectional health surveys, which resemble the actual screening setting. In the present study, we aimed to detect known colorectal ctDNA markers in plasma samples from participants of The Trøndelag Health Study (HUNT), up to 2 years prior to the clinical diagnosis of colorectal cancer.

## Methods

### Study design

This is a nested case–control study based on the Trøndelag Health study (HUNT). HUNT is one of the largest longitudinal population health studies ever performed. The HUNT Research Centre has collected data in four cross-sectional surveys: HUNT1 (1984–1986), HUNT2 (1995–1997), HUNT3 (2006–2008), and HUNT4 (2018–2019). The entire adult population ≥ 20 years old in Nord-Trøndelag county was invited to give a blood sample in the third wave of the Trøndelag Health Study (HUNT3), where of ~60,000 participated [16]. Through linkage between HUNT and the Cancer Registry of Norway (CRN), we included as cases all subjects diagnosed with colorectal adenocarcinoma ≤24 months after giving a blood sample in HUNT3. CRC was identified according to the International Classification of Diseases, 10th edition [ICD-10]: C18-20 (excluding C18.1 Appendix [ICD-7 code 153.6]; morphological codes according to the International Classification of Diseases for Oncology, 3rd edition [ICD-O-3]: 8140, 8144, 8210, 8211, 8255–8263, 8480-8481, 8490, 8510, 8570-8574, 6900, 6999, 8000–8020 (excluding NET 8041 and 8240–8246, carcinoid 8249, 8936). Among the subjects participating in HUNT3, the controls were matched 1:1 by sex and age (+/− 1 year) to the cases and were never diagnosed with CRC in the CRN (1956-31.12.2017). Other cancer diagnoses prior to or after participation were not excluded.

### Outcome and predictor variables

We defined the outcome as being diagnosed with CRC or not, within the 24 months following participation in HUNT3. A panel of promising methylated regions within 20 different genes detected in early diagnosis of CRC were selected based on systematic reviews [6, 7, 12, 13] and outstanding single publications [9, 17, 18]. Information about CRC Stage (I–IV according to the American Joint Committee on Cancer (AJCC) staging system) [19], tumour/node/metastasis (TNM) classification (AJCC) [20], haemoglobin (Hb)-level and carcinoembryonic antigen (CEA) level at diagnosis was extracted from the patients’ medical records. Date of diagnosis, diagnosis details, tumour localisation and morphology, as well as information about other cancer diagnoses were given by the CRN. Information about sex, age, body mass index (BMI), smoking (in pack-years) and diabetes was registered at the time of participation in HUNT.

### Blood samples

All blood samples were obtained by a skilled technician at the time of participation in HUNT, transported to HUNT Research Centre at 4 °C, centrifuged at 6 °C for 10 min at 2500 × *g* and aliquoted within 24 h after venepuncture. The EDTA plasma aliquots were stored at minus 80 °C for future use. For the present study, all frozen samples were given a unique ID-number (phenotypes blinded to assay operators) before being couriered to Aalborg University Hospital for the methylation analyses, where plasma specimens were randomly processed.

### Analysis of methylated promoter regions in ctDNA

Two targeted markers were designed within each gene of interest. All primer and probe sequences along with amplicon sizes and detailed PCR descriptions are presented in Supplementary Methods and Supplementary Tables 1 and 2. The method for DNA extraction and methylation analysis was based on a rapid bisulphite treatment of cell-free DNA extracted from plasma samples with subsequent 2-step PCR detection, according to a protocol previously published [21]. In brief, plasma nucleic acids were extracted using the easyMAG™ platform (NucliSens® [bioMerieux SA, France]) according to the manufacturer’s instructions. For the extraction, 2 × 900-μl EDTA plasma was used, and purified nucleic acids were eluted in 2 × 25-μl elution buffer. Five microliters were used for quantitation of extracted DNA, and the remainder was deaminated by mixing with 90-μl deamination solution and deaminated for 10 min at 90 °C, followed by purification using EasyMag and elution in 25 μl 10 mM KOH.

The method was not quantitative and hence there were no reference intervals, ranges or CVs (coefficient of variation). Units of measurement was cycle threshold (Ct) values. The naturally hemimethylated *MEST1* was used as a quality control parameter (reference gene for methylation), to ensure the correct assessment of the effect of methylation changes in the other methylation markers. The corrected Ct-value was used for calculations, where a correction factor was used to account for differences in PCR effectivity in different promoter sequences. The correction factor was calculated as mean Ct-value of eight replicates of global methylated DNA (EpiTect methylated human control DNA) minus mean Ct of *MEST1*.

### Statistical analyses

The outcome variable (CRC/no CRC) and the following potential predictor variables were handled as binary: methylated/not methylated for each of the biomarkers, sex (male/female), diabetes/no diabetes and other cancer/no other cancer. The following potential predictor variables were handled as continuous: age, smoking (pack-years), BMI, Hb- and CEA-level. The number of pack-years was missing for 16 of the 212 subjects in the study. These missing values were replaced by the median pack-years of the entire population (7.0 pack-years). Similarly, one of the 212 subjects had no BMI calculated and the missing value was replaced by the median of the entire population (BMI 27.1 kg/m^2^). Comparisons of clinical variables and methylation status between cases and controls were made by Χ^2^ test for categorical variables and *t* test for continuous variables (two-sided).

SPSS split the samples into a test set (70% of samples) and a validation set (30% of samples), by simple randomisation based on the personal identification number. The test set was used for analyses leading to the selection of a marker panel, whereas the validation set was used exclusively to determine if the results of the selected marker panel could be reproduced in an independent sample. In the test set, the cut-off corrected Ct-value to define a sample as methylated/not methylated for each marker was defined as the value that maximised the Youden index [22]. All samples with values less than or equal to this cut-off were deemed positive (methylated). All samples with Ct-values greater than the cut-off value, Ct-values > 40 or missing values were deemed negative (unmethylated). To rule out the markers hampered by sporadic background methylation, only markers with an AUC >0.5 were considered of interest for diagnostic purposes and further analysed in the present study (Supplementary Table 3). For gene markers *AGBL4*, *BCAT1*, *IKZF1*, *SEPT9*, *SFRP1*, *VIM* and *WNT5A* sense promoter sequences were used. For *BMP3*, *FLI1*, *NDRG4*, *NPTX2*, *SDC2*, *SFRP2*, *SLC8A1* and *ZNF331* antisense promoter sequences were used. The association between the outcome variable and the predictor variables was analysed by binary logistic regression in univariable and multivariable models adjusting for potential confounders [23]. Sensitivity, specificity, positive predictive value (PPV), negative predictive value (NPV) and accuracy (Acc), as well as receiver operating characteristic (ROC) curves and area under the ROC curve (AUC) were estimated for each of the single markers. For ROC curves, the probability of the multivariable logistic regression was used as the test variable to plot the covariate-adjusted ROC curves (AROC) [23]. The putative predictors of CRC with a significance level below 0.1 (*P* < 0.1) in the AROC analyses were considered possible candidates for a diagnostic methylation panel and used as a panel of markers in the test set. Ultimately, the resulting panel was tested in the validation set. All statistical analyses were done with the statistical software packages SPSS^®^ version 28.0.1.0.

## Results

### Study population

Plasma from 212 individuals (106 cases and 106 controls) was sent for ctDNA analysis (see Supplementary Fig. 1). The 212 samples were randomly divided into two groups, one test set containing 70% of the samples (*n* = 143) and one validation set containing 30% of the samples (*n* = 69). The test set and the validation set did not differ regarding sex, age, BMI, diabetes, Hb, CEA, TNM-stage or AJCC stage (*P* > 0.05). In the validation group, cases had smoked more than controls (mean pack-years 16.0 versus 6.5, *P* < 0.05) (Table 1).

**Table 1:** Description of the study population.

	Test set			Validation set		
	Colorectal cancer	Healthy controls	*P* value	Colorectal cancer	Healthy	*P* value
*N* (%)	72 (49.7%)	71 (50.3%)		34 (49.3%)	35 (50.7%)	
Female	40 (55.6%)	37 (52.1%)	0.680	15 (46.9%)	19 (51.4%)	0.711
Male	32 (44.4%)	34 (47.9%)	0.680	17 (53.1%)	18 (48.6%)	0.711
Age, mean (SD)	69.8 (10.3)	69.7 (10.6)	0.857	69.5 (10.1)	69.5 (9.1)	0.483
Pack-years, mean (SD)	14.7 (16.4)	12.1 (16.7)	0.899	16.0 (19.2)	6.5 (9.6)	**0.009**
BMI, mean (SD)	27.3 (4.1)	27.3 (4.1)	0.568	27.6 (4.2)	27.6 (3.5)	0.383
Diabetes	7 (9.7%)	3 (4.2%)	0.198	1 (2.9%)	1 (2.9%)	0.983
Hb, mean (SD)	12.2 (2.2)			12.4 (2.0)		
CEA, mean (min–max)	67.5 (1.0–1068.0)			68.5 (1.0–1848.0)		
Median	4.0			3.0		
Tumour localisation, *n* (%)						
RCC	30 (41.7%)			15 (44.1%)		
LCC	22 (30.6%)			11 (32.4%)		
RC	20 (27.8%)			8 (23.5%)		
Tumour stage, *n* (%)	54 (75.0%)			30 (88.2%)		
T1	0 (0%)			2 (6.7%)		
T2	11 (20.4%)			5 (16.7%)		
T3	35 (64.8%)			20 (66.7%)		
T4	8 (14.8%)			3 (10.0%)		
Node stage, *n* (%)	57 (79.2%)			29 (85.3%)		
N0	40 (70.2%)			14 (48.3%)		
N1	12 (21.1%)			8 (27.6%)		
N2	5 (8.8%)			7 (24.1%)		
Metastasis stage, *n* (%)	68 (94.4%)			33 (97.1%)		
M0	53 (77.9%)			28 (84.8%)		
M1	15 (22.1%)			5 (15.2%)		
Stage (AJCC)						
I	6 (8.3%)			2 (5.9%)		
II	37 (51.4%)			15 (44.1%)		
III	13 (18.1%)			12 (35.3%)		
IV	16 (22.2%)			5 (14.7%)		

*SD* standard deviation, *BMI* body mass index (kg/m^2^), *Hb* haemoglobin (g/dl), *CEA* carcinoembryonic antigen (µg/l), *RCC* right colon cancer, *LCC* left colon cancer, *RC* rectal cancer, *T* tumour, *N* Node, *M* metastasis, *AJCC* American joint committee on Cancer. Bold value = *p* < 0.05.

*Χ*^2^ test was used for categorical variables and *t* test for continous variables.

### Detection of ctDNA methylation markers

The number and percentage of cases and controls deemed positive for each of the putative diagnostic markers in the test set are presented in Supplementary Table 4. By chi-square analyses, the markers with significant associations to disease status were *FLI1, IKZF1, SFRP2* and *VIM* (*P* < 0.05) (Supplementary Table 4). However, as age, sex, BMI and smoking are all associated with both methylation status [24–26] and with CRC risk [27], these factors were considered confounders and needed to be adjusted for [23]. Univariable and multivariable binary logistic regression analyses were performed to investigate whether the methylation status of any of the single markers was a significant, independent predictor of colorectal cancer. The three markers *IKZF1* (OR 3.54, 95% CI 1.19–10.49), *FLI1* (OR 3.07, 95% CI 1.12–8.45) and *SFRP2* (OR 2.69, 95% CI 1.11–6.49) were all independent predictors (*P* < 0.05) after adjusting for age, sex, BMI and smoking (Table 2). None of the covariates sex, age, smoking and BMI was significantly associated with colorectal cancer (Table 2).

**Table 2:** Regression models.

	CRC vs healthy univariate (unadjusted)			CRC vs healthy multivariable (adjusted^*^)		
Variables for inclusion	OR	95% CI	*P* value	OR	95% CI	*P* value
*AGBL4*	2.163	0.974–4.805	0.058	2.036	0.903–4.591	0.086
*BCAT1*	3.136	0.611–16.096	0.171	3.133	0.593–16.543	0.179
*BMP3*	6.364	0.746–54.279	0.091	6.451	0.737–56.482	0.092
*FLI1*	3.095	1.134–8.448	**0.027**	3.069	1.115–8.449	**0.030**
*IKZF1*	3.474	1.189–10.151	**0.023**	3.536	1.193–10.485	**0.023**
*NDRG4*	1.550	0.742–3.239	0.244	1.567	0.744–3.299	0.237
*NPTX2*	2.163	0.974–4.805	0.058	2.143	0.954–4.815	0.065
*SEPT9*	2.441	0.605–9.845	0.210	2.452	0.601–10.003	0.211
*SDC2*	2.833	0.720–11.151	0.136	2.867	0.721–11.400	0.135
*SFRP1*	2.163	0.974–4.805	0.058	2.066	0.920–4.643	0.079
*SFRP2*	2.837	1.195–6.732	**0.018**	2.687	1.112–6.493	**0.028**
*SLC8A1*	2.033	0.864–4.783	0.104	2.014	0.847–4.788	0.113
*VIM*^†^	–	–	–	–	–	–
*WNT5A*	1.523	0.411–5.644	0.529	1.490	0.394–5.642	0.557
*ZNF331*	1.719	0.819–3.608	0.152	1.676	0.781–3.596	0.185
Age	1.001	0.970–1.033	0.964	–	–	–
Smoking	1.010	0.990–1.030	0.350	–	–	–
BMI	0.998	0.920–1.082	0.952	–	–	–
Sex	0.871	0.451–1.681	0.680	–	–	–

^*^Adjusted for age, sex, BMI and smoking. Bold value = *p* < 0.05.

^†^It was not possible to perform regression on *VIM* due to only one outcome (cases (*n* = 4), controls (*n* = 0)).

### Diagnostic properties of single markers and panel

**Fig. 1:**
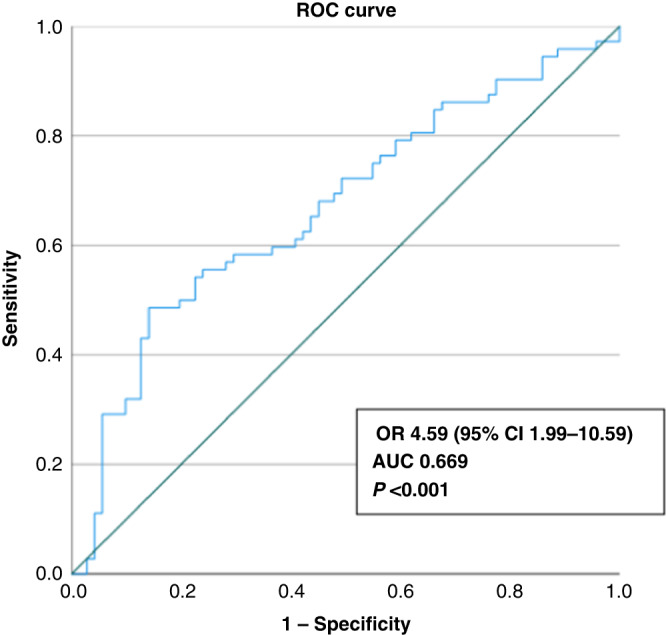
ROC analysis HUNT-CRC_d_ panel. ROC analysis for separating healthy controls from the patients diagnosed with colorectal cancer within the subsequent 24 months, based on having two or more markers within the panel of eight (adjusted for sex, age, BMI and smoking).

**Fig. 2:**
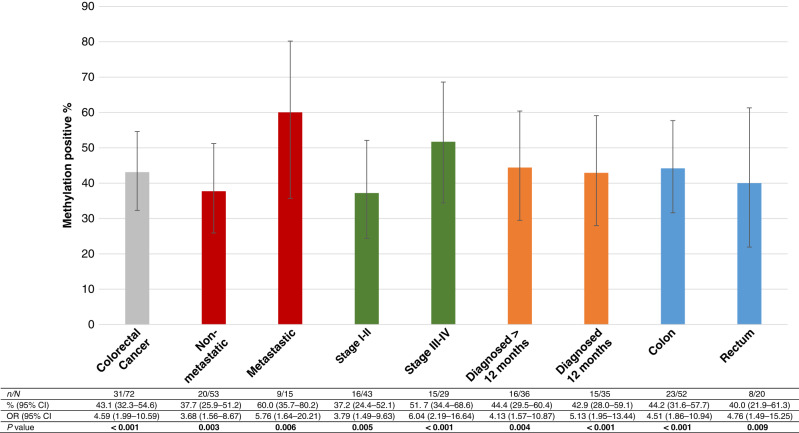
Detection of methylated DNA by clinical phenotype. A specimen was deemed positive if at least two of eight markers in the panel were positive. OR, odds ratio of binary logistic regression or multinomial logistic regression adjusted for sex, age, BMI and smoking, with controls as reference category. 95% CI by Wilson score.

The sensitivity, specificity, PPV, NPV and accuracy of each of the single markers are presented in Table 3. The most sensitive markers were *NDRG4* (62.5%) and *WNT5A* (51.4%). The most specific single markers were *AGBL4, BMP3, FLI1, IKZF1, NPTX2, SFRP1, SFRP2, SDC2, SLC8A1* and *VIM* (specificity >70%). As expected, based on previous studies showing that no single ctDNA methylation marker is considered adequately sensitive and specific for cancer screening, we found it important to investigate the combined effect of the most specific and significant markers in a panel. *BMP3, FLI1, IKZF1, NPTX2, SFRP1, SFRP2, SLC8A1* and *VIM* were combined into the HUNT-CRC diagnostic panel (HUNT-CRC_d_), considered positive if two or more of the eight markers were present (Supplementary Fig. 2A). A positive panel inferred an odds ratio of 4.59 (95% CI 1.99–10.59, *P* < 0.001) of being clinically diagnosed with colorectal cancer within the subsequent 24 months with AUC of 0.669 (Fig. 1). The detection rate of the panel was higher among the subjects with metastatic disease (60.0%) than among the subjects with non-metastatic disease (37.7%) and increased with increasing AJCC stage of disease (Fig. 2). The odds ratio of being diagnosed with colorectal cancer within 12 months was 5.13 (95% 1.95–13.44), whereas the odds ratio of being diagnosed 12 months or more after blood sampling was 4.13 (95% CI 1.57–10.87) (Fig. 2). Detection rate among rectal cancer was 40%, whereas the rate in colon cancer was 44% (Fig. 2). We also investigated whether any predictor was associated with assay positivity in the healthy controls, but no such predictor was found (Supplementary Table 5). The distribution of other malignancies in cases and controls is presented in Supplementary Table 6. The number and distribution of positive markers within the HUNT-CRC_d_ panel for cases and controls is shown in Supplementary Fig. 2A, B. The precision of the HUNT-CRC_d_ panel is shown in Supplementary Table 7.

**Table 3:** Diagnostic properties of single markers and panel.

	AROC analysis^†^			Sensitivity CRC		Specificity CRC		PPV	NPV	Acc
	AUC	95% CI	*P* value	%	95% CI	%	95% CI	%	%	%
*AGBL4*	0.572	0.477–0.667	0.136	40.3	39.9–40.6	77.5	77.2–77.8	64.4	56.1	58.7
*BCAT1*	0.570	0.476–0.664	0.147	43.1	42.7–43.4	64.8	64.4–65.1	55.4	52.9	53.8
*BMP3*	0.592	0.498–0.685	0.059	41.7	41.3–2.0	76.1	75.7–76.4	63.8	56.3	58.7
*FLI1*	0.590	0.497–0.684	0.063	38.9	38.5–39.2	76.1	75.7–76.4	62.2	54.5	57.3
*IKZF1*	0.603	0.510–0.696	**0.034**	43.1	42.7–43.4	78.9	78.6–79.2	67.4	57.7	60.8
*NDRG4*	0.570	0.476–0.665	0.147	62.5	62.1–62.9	47.9	47.5–48.3	54.9	55.7	55.2
*NPTX2*	0.607	0.514–0.700	**0.027**	48.6	48.2–49.0	74.6	74.3–75.0	66.0	58.9	61.5
*SEPT9*	0.565	0.470–0.660	0.177	48.6	48.2–49.0	69.0	68.7–69.4	61.4	57.0	58.7
*SDC2*	0.565	0.470–0.660	0.179	41.7	41.3–42.0	70.4	70.1–70.8	58.8	54.3	55.9
*SFRP1*	0.596	0.503–0.690	**0.047**	40.3	39.9–40.6	77.5	77.2–77.8	64.4	56.1	58.7
*SFRP2*	0.600	0.506–0.693	**0.039**	31.9	31.6–32.3	85.9	85.7–86.2	69.7	55.5	58.7
*SLC8A1*	0.589	0.496–0.683	0.065	45.8	45.5–46.2	73.2	72.9–73.6	63.5	57.1	59.4
*VIM*	0.595	0.502–0.688	0.051	45.8	45.5–46.2	70.4	70.1–70.8	61.1	56.2	58.0
*WNT5A*	0.568	0.474–0.663	0.159	51.4	51.0–51.8	60.6	60.2–0.9	56.9	55.1	55.9
*ZNF331*	0.570	0.475–0.665	0.147	50.0	49.6–50.4	66.2	65.8–66.5	60.0	56.6	58.0
HUNT-CRC_d_*	0.669	0.580–0.759	**<0.001**	43.1	42.7–43.4	85.9	85.7–86.2	75.6	59.8	64.3
HUNT-CRC_d_* val	0.680	0.553–0.806	**0.010**	47.1	46.5–47.6	77.1	76.7–77.6	66.7	60.0	62.3

*AUC* area under the curve, *AROC* adjusted receiver operating characteristic curve, *Acc* accuracy, *CRC* colorectal cancer, *PPV* positive predictive value, *NPV* negative predictive value, *95% CI* 95% confidence interval, *HUNT-CRC*_*d*_ panel of the markers *BMP3, FLI1, IKZF1, SFRP1, SFRP2, SLC8A1, VIM* and *NPTX2, val* validation set. Bold value = *p* < 0.05.

*Positive panel = at least two of the eight markers present, val, validation set, ^†^adjusted for age, BMI, sex and smoking.

## Discussion

To our knowledge, this is one of the first studies systematically detecting known colorectal ctDNA methylation markers in plasma up to 2 years prior to the clinical diagnosis of CRC, within an unselected, cross-sectional population study resembling the screening setting.

Interestingly, we found that four of the markers best suited to distinguish cases from controls in our study were the already well-known colorectal cancer markers *IKZF1*, *SFRP1*, *SFRP2* and *VIM* [6, 7]. Hence, our study validates the findings of previous studies, but now also in an unselected population upstream of the clinical diagnosis [9, 28–31]. The latter represents a big step needed before clinical implementation of ctDNA in colorectal cancer screening [6]. In addition to *IKZF1*, *SFRP1*, *SFRP2* and *VIM*, this study demonstrates that *BMP3*, which so far has been used mostly as a stool-based biomarker, also has value as a plasma biomarker. To our knowledge, only one small previous study has investigated the value of *BMP3* and concluded that the sensitivity of this marker alone was not sufficient for detection of CRC [32]. Furthermore, the markers *FLI1*, *SLC8A1* and *NPTX2* in our panel have also been demonstrated to be of interest in previous studies [7, 12, 18]. Consequently, *BMP3*, *FLI1*, *IKZF1*, *NPTX2, SFRP1*, *SFRP2 SLC8A1* and *VIM* could now be considered as ready for evaluation in prospective phase 4 screening studies [6].

The minimal invasiveness and simplicity of this methylation-specific PCR-based ctDNA panel (for example relative to the more resource-intensive next-generation sequencing), makes it attractive to use in clinical settings where the sensitivity is adequate. Possible areas of use could be family members of colorectal cancer patients (increased sensitivity when repeating the panel annually in the same individual), and as a supplement among those refusing colonoscopy or iFOBT/FIT [33]. In line with previous research, our investigation confirm that for screening purposes it is the most specific markers (i.e., those that are detected only at low levels in healthy controls) that are most valuable [6]. As methylation is a normal cell regulatory event and the majority of cfDNA is derived from normal cells form various tissues and organs, and also from white blood cells (WBCs), it is important that tumour-specific DNA methylation markers show rare to no methylation in normal tissues and WBCs. Differently methylated regions within markers such as *BCAT1*, *SEPT9* and *IKZF1* have previously been shown to have ignorable levels of methylation in WBCs and high levels in colonoscopy-confirmed patients with colorectal cancer [34]. This feature would also need to be investigated for *BMP3*, *FLI1*, *NPTX2, SFRP1*, *SFRP2*, *SLC8A1* and *VIM* before testing our suggested panel in prognostic screening populations.

Many studies have used an arbitrary cut-off Ct-value (i.e., 40 cycles or 45 cycles) to deem a sample as methylated or not, while some have classified all samples with Ct-values as methylated. This study use the Youden index to define a cut-off Ct-value for each biomarker. The Youden index maximises the sensitivity and specificity of the test, and could therefore be beneficial in a diagnostic setting. The number of cycles required for detectable amplification of colorectal ctDNA markers is dependent on a long list of factors beyond the amount of tumour DNA present in the specimen. Ct-values are influenced by pre-analytic variables such as efficiency of the blood sampling, storage and transport, analytic variables including DNA extraction efficiency, analysis platform used, nature of the target marker, design of the primers/probes and finally by the clinical variables such as stage of disease and presence of other diseases including other cancer. If researchers control their experiments, Ct-values can be used in one experiment as guide to compare one result to another, but the comparison of Ct-values between labs or experiments is not meaningful because of the abovementioned variations. This is a hindrance to commercialisation of methylation-based tests for CRC screening, but not prohibitive, as demonstrated by tests already available in the market (i.e., EpiProcolon [7], Colvera [35]).

There are several limitations in this study. First, when blood sampling was performed, ctDNA analyses was not yet planned, hence the sampling was not optimised for such analyses. One main problem could be cell lysis and hence contamination of ctDNA in plasma with DNA from lysed blood cells. As we selected for further analyses only markers with an AUC >0.5, markers found in both healthy controls and cases (possibly due to lysis) were largely ruled out. In addition, decay of ctDNA due to lengthy storage is well-known and the plasma samples in this study were prepared 11–13 years prior to ctDNA analyses [36]. However, if ctDNA decay was present in our study, we believe our findings would be easy to reproduce in fresh plasma samples, possibly giving an even higher sensitivity than our findings. Second, we observed a high false-positive rate in healthy controls increasing with higher Ct-values. This illustrates the well-known aspect that by running many PCR-cycles unspecific methylation detection occurs and one needs to place the Ct-cut-off values low enough to rule this out. Third, we observed false-negative results among cases, which would be problematic in screening. However, the volume of plasma used in this study was only 2 × 900 µl and one would expect that using plasma from a 10-ml EDTA blood sample (~4.5 ml plasma) would increase the sensitivity and value of the test dramatically. Because of the very small amounts of ctDNA, 1.8 ml plasma may not contain one full copy of the cancer genome, meaning that some markers may be present (and detectable), whereas others are not. Finally, the case–control design has been considered prone to bias in diagnostic studies. However as the cases and controls were not recruited specifically for this study and no strict inclusion or exclusion criteria were used, the risk of bias was minimised.

A strength of this study is that the controls are very likely true controls, as the observation time after blood sampling was long (9–11 years), all Norwegian inhabitants have the same access to health care and the quality of the CRN is high. The quality of plasma samples in HUNT Biobank is high [37]. Compared to other studies, the present study population is probably closer to a true screening population and hence, generalisable to this setting. Firstly, in studies where cases are included only at the time of diagnosis (often symptomatic), early-stage disease will be under-represented. Secondly, in studies where possible controls are excluded due to age, other cancer or comorbidities, the included ones do no longer resemble a screening population. The HUNT population, from which our controls were selected, is known to have the same or higher occurrence of other diseases than the general population [16]. These facts make the sensitivity and specificity of this study more modest than other studies, but also more transferable to real-life screening. However, selection bias due to differences between participants and non-participants of the HUNT study can not be ruled out [38]. Finally, we have used the STARD guidelines to increase the research value in a field hampered by a lack of clinical translation much due to weaknesses in methodological design and reporting of findings [39].

In conclusion, detection of known methylated ctDNA markers for CRC is possible up to 2 years prior to the clinical diagnosis. As suggested by others, this study supports the hypothesis that some patients could be diagnosed earlier, in a possible curable setting, if ctDNA detection was implemented as part of the CRC screening programme. To evaluate this hypothesis further, prospective studies and cost analyses addressing the overall benefit of ctDNA in CRC screening are needed.

## Supplementary information

Supplementary materials

STARD

## Data Availability

Data may be obtained from a third party and are not publicly available. The data that support the findings of this study are deidentified participant data as well as biological materials, available from HUNT upon application (https://www.ntnu.no/hunt, e-mail: kontakt@hunt.ntnu.no). Restrictions apply to the availability of these data, which were used under licence for this study.
